# Navigating Borders: Language, Identity, and Schooling Among Return Migrant University Students

**DOI:** 10.3390/bs16030424

**Published:** 2026-03-14

**Authors:** Mónica L. Jacobo Suárez, Colette Despagne Broxner

**Affiliations:** 1 Department of Politics and Culture, Universidad Autónoma Metropolitana Xochimilco, Mexico City 04960, Mexico; 2 Instituto de Ciencias Sociales y Humanidades “Alfonso Vélez Pliego”, Benemérita Universidad Autónoma de Puebla, Puebla 72000, Mexico; colette.despagne@correo.buap.mx

**Keywords:** return migration, monolithic education systems, language maintenance, transpositioning, transnationalism

## Abstract

Grounded in transnationalism and poststructuralist theories of identity, this study examines how educational trajectories shape language maintenance and identity development among return migrant students in Mexican universities who were educated across the United States (U.S.) and Mexico. Using a multi-sited qualitative case study design, we draw on in-depth interviews and reconstructed educational histories, analyzed comparatively across generational cohorts, age of return, and complete educational trajectories. The analysis identifies four distinct educational profiles, illustrating how different configurations of transnational schooling create differentiated conditions for language use, bilingual development, and identity negotiation. Findings show that while institutional contexts often reflect monoglossic ideologies, students strategically mobilize their transnational linguistic and cultural resources to navigate these settings. The study underscores the need for policy and pedagogical frameworks that recognize heterogeneous educational pathways and treat transnational repertoires as assets rather than deficits.

## 1. Introduction

“*I left for the U.S. when I was practically 7 months old, so I didn’t know how to speak Spanish*.” This quote from Marian, a young return migrant to Mexico, encapsulates the core dilemma this article addresses: the profound clash between transnational life trajectories and national education systems utterly unprepared to receive them. Her words personalize a growing reality, immediately anchoring the abstract problem of reintegration in a human struggle with language, belonging, and identity.

Mexico has undergone a significant demographic shift, transitioning from a nation defined primarily by emigration—understood here as outward migration from Mexico, primarily to the U.S.—to one increasingly characterized by return migration and population flows in transit (Escobar & Masferrer, 2021). This new landscape includes a rising number of children, adolescents, and young adults of Mexican origin who, like Marian, belong to the so-called 1.5 generation—youth who migrated at an early age and were largely educated in the destination country— and have transitioned from living in the U.S. to now residing in Mexico. Hailing from mixed-status families, their “return” is often not experienced as a homecoming but rather as a migration to a country that is paradoxically both familiar and alien due to their socialization in U.S. schools (Jacobo & Despagne, 2022).

The central problem examined in this article is the profound lack of support programs for students with transnational backgrounds within the Mexican educational system, a system characterized by its monolithic—that is, monolingual and monocultural—nature (Zúñiga, 2025; Vargas, 2022; Despagne, 2024). We understand transnational students as those who have developed academic trajectories across two or more national educational systems—such as Mexico and the U.S.—regardless of their place of birth or citizenship (Zúñiga & Hamann, 2008). To analytically ground their experiences, we draw on the concept of transnationalism, which we define as the flows and exchanges that occur across the U.S.-Mexico border among the study’s participants, encompassing the movement of people, ideas, information, and goods. More specifically, we focus on a transnationalism “from below,” referring to the mundane, everyday grassroots activities of participants (Smith, 1998).

The Mexican education system that transnational students encounter is structured around a single dominant linguistic code—Mexican Spanish—and a singular cultural framework centered on Mestizo identity. It operates under the assumption that there is only one “normal” and “correct” language and culture through which to be recognized as a “real” Mexican, expecting all students to conform to this standard. As a result, students whose dominant language is not Spanish are placed at a significant disadvantage, particularly given the absence of Spanish as a Second Language (L2) programs that would support their academic integration through scaffolded instruction. With the exception of the Bilingual Intercultural Education System—which serves Indigenous students at the elementary level and largely functions as a transitional model toward Spanish rather than a fully bilingual program—Mexico has no public bilingual or dual-language schools. Instead, the public system offers only English as a Foreign Language (EFL) instruction at the basic levels.

This stands in stark contrast to the U.S. education system. Although the U.S. similarly pursues monolingualization, it provides scaffolded instruction that enables students to learn English. In Mexico, by contrast, transnational students must develop their own strategies for acquiring Spanish. Within the school context, because they are Mexican, they are expected to understand, speak, read, and write in Spanish on par with any lifelong native speaker.

This institutional void exposes transnational students to a series of challenges that extend beyond the academic, spilling into the linguistic, identitarian, and social dimensions, frequently generating significant social suffering (Jardón & Macías Suárez, 2024; Rodríguez-Cruz, 2022). This study focuses on return migrants with transnational educational experiences, a group that can be described as “hypervisible and yet invisible”. They are hypervisible due to their accented Spanish, hybrid cultural codes, and English proficiency^1^, while simultaneously invisible, first, because they look like any other Mexican, and second, because educational systems make them invisible by insisting on homogenization and by ignoring their rich transnational funds of knowledge (Zúñiga & Román, 2022).

Although existing research has effectively documented return migrant students’ broad linguistic barriers and systemic exclusion, it has fallen short of explaining the variation in their experiences and identifying the variables that produce divergent outcomes. This study addresses that gap by systematically disaggregating the diverse linguistic and identity reconfigurations among transnational students who are return migrants. We argue that a nuanced understanding requires moving beyond general patterns to examine how specific educational trajectories—shaped by age of emigration, age of return, and the sequencing of schooling across two national systems—create distinct conditions for language maintenance (the preservation and development of English and Spanish in Mexico), English attrition, and the negotiation of transnational identities. Throughout, we adopt an asset-based lens that recognizes return migrant students as resourceful transnational actors endowed with complex linguistic and cultural repertoires rather than as students positioned within a deficit framework.

With this goal, this paper aims to examine how the age of emigration to the U.S. and the age of return to Mexico—and consequently, the sequence of their transnational schooling—critically influence their linguistic proficiency in Spanish and English, as well as their complex processes of identity reconfiguration. Crucially, this analysis does not focus solely on deterministic theoretical relations but seeks to highlight the remarkable agency that young return migrants deploy to navigate and resist adverse contexts.

To address these puzzles, the remainder of the article proceeds as follows. Section 2 establishes the empirical and institutional context, defining the return migrant student population and situating the Mexican educational system as a contested terrain shaped by monolithic ideologies. Section 3 outlines our integrated theoretical framework for understanding return migrant students’ linguistic and identity trajectories. Section 4 describes our qualitative, multi-sited methodology and analytical strategy. Section 5 presents the findings, demonstrating how educational trajectories shape divergent linguistic and identity outcomes. Finally, Section 6 discusses the theoretical and policy implications, highlighting students’ agentive negotiations in the face of systemic exclusion and calling for transdisciplinary approaches to transform Mexican educational spaces.

## 2. Return Migrant Youth in Mexico’s Shifting Educational Landscape

### 2.1. Understanding Return Migrant Youth and Children

Mexico’s migration landscape has undergone a significant transformation, evolving from a nation of emigration to one increasingly defined by return migrants and transit flows (Escobar & Masferrer, 2021). Within this shift, a distinct and growing population has emerged: children and youth of Mexican origin who circulate, in some cases multiple times, between the U.S. and Mexico. This study uses return migrants to refer to Mexican-origin immigrants who move from the U.S back to Mexico. In particular, we focus on young returnees who were, at the moment of the study, active students at a public university in Mexico.

These young people are not first-generation migrants, nor are they monolingual or monocultural Mexicans; they are individuals marked by transnational mobility, often possessing hybrid national affiliations and transnational educational experiences (Hamann & Zúñiga, 2011). Depending on the context and temporal conditions, they may identify more strongly with one side of the continuum or the other. This is a substantial population, with nearly 1.5 million students with ties to the U.S. now in Mexican schools, representing about 5% of the scholastic population (Jacobo & Jensen, 2018). Their return to Mexico, whether voluntary or forced, marks a critical turning point in their lives.

As Zúñiga (2025) highlights, transnational students are not merely passive victims of macroeconomic forces but active strategists, often supported by parents who deliberately navigate institutional barriers to advocate for their children’s education and bilingual futures. Yet this agency is frequently constrained at the moment of return. Most participants report having had “no say at all” in the decision to migrate back to Mexico, and some expressed a clear desire to remain in the United States (Despagne & Manzano-Mungía, 2020). In this sense, the return process is often experienced as externally imposed rather than self-determined.

Over time, agency becomes more consciously enacted and strategically directed. Many participants mobilize their bilingual repertoire in shaping their educational and professional pathways. This is particularly evident in career decisions: several leverage their bilingualism to enter fields such as English language teaching, viewing it as a stable trajectory that capitalizes on their transnational linguistic capital (Mora-Pablo et al., 2025). Taken together, these trajectories illustrate how structural constraint and agentive repositioning coexist. Transnational students thus represent a distinctive demographic that challenges monolithic conceptions of national identity and belonging, while illuminating the complex interplay between institutional structures and individual strategies in contemporary return migration to Mexico.

### 2.2. The Mexican Educational System as a Contested Terrain

For young returnees, the Mexican educational system represents a primary site of (re)integration but also of conflict and identity negotiation. Existing literature reveals that Mexican schools often function as a “contested terrain,” where institutional structures can either reinforce exclusionary national ideologies or, potentially, provide space for multilingual identities. A consistent finding across studies is the prevalence of administrative and bureaucratic barriers in Mexico. Despite policy changes, programs for return migrant students depend on political convenience rather than implementable infrastructures, creating significant obstacles for returnees (Jacobo, 2017; López et al., 2018; Ruíz & Valdez, 2024). In addition, there is a lack of federal support; the Mexican Ministry of Education offers no specific programs to help return children linguistically or cognitively adapt to their new school context, rendering them officially “invisible” to the system (Despagne & Manzano-Mungía, 2020).

Furthermore, research shows that Mexican institutions reproduce an “ideology of mestizaje” that promotes a fixed, monolingual and monocultural ideal of the “real” Mexican citizen (Despagne, 2024). In classrooms, Spanish is imposed as the hegemonic language, foreclosing other linguistic realities (Nava et al., 2017). Consequently, return students are expected to display the same Spanish repertoires and knowledge of complex Mexican social structures as lifetime nationals, with little recognition of their migration trajectories (Despagne & Manzano-Mungía, 2020) or their transnational funds of knowledge—historically accumulated family and community resources and competencies (González et al., 2005; Zúñiga & Román, 2022). This transnational linguistic and cultural capital is often devalued or seen as a threat, exposing students to discrimination and emotional strain (Jardón & Macías Suárez, 2024; Mora-Pablo, 2021). These dynamics are reflected in the narratives collected by Mora-Pablo et al. (2025), where participants recall bullying by Mexican teachers—one even threatened with being sent back to kindergarten for not knowing Spanish—while contrasting these experiences with more supportive interactions in U.S. schools.

The result is a system that, as Cortez and Hamann (2014) argue, is “lacking strategies to serve them and integrate them while respecting and valuing their diverse linguistic and identity characteristics.” While some studies have proposed critical, plurilingual, innovative, and inclusive pedagogical models (Parra & Rodríguez Cruz, 2025; Despagne & Buitrón, 2026), the broader body of literature portrays an educational system that is largely unprepared for—and often resistant to—the transnational realities of return migrant students. This is particularly true at the university level, which remains an understudied arena within this complex integration process. English Language Teaching (ELT) programs may be considered a partial exception at the higher education level (Mora-Pablo et al., 2025; Frausto-Hernandez, 2025).

The experiences of this migrant youth give rise to several pragmatic and theoretical puzzles that our study directly addresses.

## 3. Theoretical Framework: Navigating the Complexities of Return

The educational journeys of return youth within Mexico can be understood as navigating a series of challenges that emerge at the intersection of transnational life trajectories and national institutions unready to accommodate them. We address these challenges by integrating three interconnected theoretical pillars: (1) the language-identity link, (2) the ideological battleground of language and agency, and (3) the negotiated process of identity formation.

### 3.1. Deconstructing the Language-Identity Link: A Generational Cohort Approach

This section challenges the assumed direct link between linguistic proficiency and national identity during the emigration process—that is, in the destination country. While one might expect fluency to dictate belonging (e.g., English mastery correlated with a U.S. identity), return youth navigate a complex reality in which their bilingualism functions simultaneously as both an asset and a source of marginalization, depending on how it is perceived in a given context, positively or negatively (Mora-Pablo & Basurto Santos, 2019; Trejo et al., 2016). Their experience is thus defined by fluid positioning along a Mexican-American continuum.

To analyze this fluidity, the lens of generational cohorts is essential (Rumbaut, 1994, 2004). Moving beyond a simple first/second-generation dichotomy, this framework distinguishes returnees by their age of migration. These cohorts are typically defined as:

- The 1.75 generation: Those who migrated in early childhood (typically before age 8).
- The 1.5 generation: Those who began schooling in their home country before continuing in the host country.
- The 1.25 generation: Those who migrated as adolescents (often age 12 or older).

From an applied linguistics perspective, the age of migration suggests tendencies in language proficiency, such as first language (L1) maintenance or attrition (Schmid, 2008, 2013). There is an inverse relationship: early migration carries a high risk of L1 erosion, while migration after puberty promotes a greater capacity for L1 retention, even in prolonged immersive contexts (Köpke & Schmid, 2004). Since language can be a primary site of identity construction (Weedon, 1997; Umaña-Taylor et al., 2014), even though not the only one, these linguistic foundations shape outcomes.

Consequently, each cohort—1.25, 1.5, and 1.75—faces distinct identity negotiations. The 1.75 generation, socialized primarily in the U.S., often retains few significant memories of Mexico, acquires English with native-like phonetics, and develops U.S. or hybrid identities (Paris et al., 2019). The 1.5 generation, as sequential bilinguals with divided schooling, more commonly navigates hybrid identities (Vargas, 2025). In contrast, the 1.25 generation, migrating as adolescents, often maintains cultural identities more closely aligned with Mexico, resembling first-generation immigrants more than the second generation (Despagne & Buitrón, 2026).

These cohorts must be understood within the broader context of transnational fluidity. Return youth defy unidirectional migration models; they are transnational students (Skerrett, 2020) shaped by sustained cross-border ties, living in what Bauman (2012) termed liquid modernity. For them, return is often not a homecoming but a phase of complex (re)integration (Jacobo, 2022), which can be involuntary due to deportation or family separation. Reintegration is also mediated by the family unit, where parents act as strategic agents who navigate institutional barriers to advocate for their children’s bilingual futures (Zúñiga, 2025).

A critical aspect of reintegration is the inversion of language status—from English-dominant in the U.S. to Spanish-dominant in Mexico. This shift forces a reevaluation of the return youth’s linguistic capital. Returnees may leverage English for professional advancement (Mora-Pablo & Basurto Santos, 2019) while simultaneously finding their Spanish competence scrutinized as a marker of (non-)belonging. This tension underscores that identity is not a simple function of ability but a matter of agency—the strategic choices individuals make within structural constraints or what Ahearn (2001) defines as “the socioculturally mediated capacity to act” (p. 112).

Furthermore, the relationship between language and identity is dialectical, not unidirectional. National identification can actively influence L1 competence. Schmid (2011) argues that language attrition can be accelerated by rejecting one’s heritage culture, while a positive ethnic identity acts as a predictive factor for L1 maintenance. For instance, the contemporary anti-immigrant climate in the U.S. may pressure families to adopt English-only practices, whereas children who may be able to construct a comfortable Mexican ethnic identity are more likely to sustain Spanish proficiency. Ultimately, within this transnational context, identity is an active negotiation, a strategic process where linguistic resources are managed amid shifting pressures of belonging, exclusion, and aspiration.

### 3.2. The Age of Return: Linguistic Agency and Educational Reintegration

While the previous section established age of emigration as an aspect that influences linguistic and cultural starting points, here we focus on the developmental timing of reintegration into the Mexican educational system and its impact on long-term agency. We argue that analyzing the *complete* educational trajectory—spanning both emigration *and* return—is essential for understanding how language, identity and schooling interconnect in the transnational experiences of young returnees, even though it is not the only element. Family language policies, language ideologies and beliefs also play an important role in language attrition and/or maintenance (Tang & Calafato, 2025), even though they are not directly addressed in this study.

Upon returning to Mexico, return youth confront an educational and social system shaped by monoglossic language ideologies. This system operates under a national “dogma of homogeneism” (Blommaert & Verschueren, 1991), rooted in historical mestizaje narratives (Vasconcelos, 1948; Bustamante, 2021). The result is a critical contradiction: while English is valued as a global commodity, its use by returnees is often stigmatized as inauthentic or pretentious. This stigma is frequently tied to raciolinguistic ideologies (Rosa & Flores, 2017)—beliefs that conflate language with racial identity to reproduce social hierarchies. Within this frame, high English proficiency becomes linked with whiteness and elite status (Despagne, 2024; Rosa & Flores, 2017), marking returnees as outsiders.

The age at which a student returns to Mexico shapes the nature of the challenges they encounter. Returnees may face academic difficulties in developing Spanish literacy, understood as the ability to identify, interpret, and communicate through written materials in Spanish across diverse social and culturally situated contexts (Barton & Hamilton, 1998). Alternatively, they may confront barriers to social integration (Trejo et al., 2023), depending on their developmental stage and prior schooling experiences (Nava et al., 2017). At this point, the limitations of traditional frameworks such as language attrition (Schmid, 2008, 2013) become evident. These models, centered on unidirectional movement and destination contexts, fail to capture the bidirectional trajectories of transnational students.

For return youth, the age of return marks not an endpoint but a pivotal transition that reactivates and recontextualizes their linguistic repertoire. Although linguistic proficiency may correlate with the language of primary schooling, this relationship is not deterministic. Heritage language research shows that competence positively correlates with strong bicultural identification (Little & Zhou, 2024), highlighting that these youth are not passive subjects seeking to “fit in” but agentive individuals strategically navigating multiple linguistic and cultural spaces.

Hence, return youth navigate Mexico’s monolingual ideologies in divergent ways. While some may assimilate and gradually lose proficiency in English, others actively resist these pressures through transpositioning—the iterative, agentive capacity to shift across social and linguistic positions. In doing so, students mobilize their plurilingual and pluricultural resources, strategically activating multiple identities and moving across the boundaries of named languages, digital media, and entrenched national ideologies. Through this process, “borders are renegotiated, circumvented, even outright rejected” (Wei & King Lee, 2023, p. 14). Such negotiation across contexts, languages, and cultures enables them to “spontaneously (re)invent themselves by orchestrating all available and accessible resources in their semiotic repertoire in response to communicative stimuli from others” (Wei & King Lee, 2023, p. 14).

Transpositioning is therefore an agentive act, as return migrant students become social actors capable of transforming communicative situations and their own social positioning. They act within their contexts to shape new future realities while navigating imposed social, temporal, symbolic, and linguistic boundaries. Accordingly, the strategic maintenance of English, the development of academic Spanish, or the covert use of Spanglish are not merely adaptive behaviors but deliberate exercises of linguistic agency. These practices constitute agentive acts of transpositioning through which students reclaim plurilingual and pluricultural belonging, converting transnational struggles into strategic skills or transnational capital (Frausto-Hernandez, 2025).

Yet this agency is unevenly distributed and deeply shaped by social class and locality. Zúñiga’s (2025) research highlights stark disparities: while some urban, middle-class families implement structured homeschool programs to maintain English, resource-poor rural families often lack such means. Even so, individual and family motivation can be decisive. The case of “Beto” (Zúñiga, 2025) illustrates this dynamic: despite living in a small Mexican town without institutional support, his desire to return to the U.S. led him to maintain his English independently through media and by teaching his brother. His proactive stance demonstrates how personal conviction can mitigate a monolingualizing environment, showing that agency operates within—and often against—powerful structural constraints.

A comprehensive analysis must therefore consider both the age of emigration, which structures linguistic foundations, and the age of return, which shapes the conditions for exercising agency. The interplay of these pivotal moments across a student’s educational trajectory ultimately influences linguistic and identificatory outcomes for return youth.

### 3.3. The University as a New Arena for Identity Negotiation

While most research on return migration concludes at the secondary level, the university constitutes a critical new phase—one marked by greater autonomy, specialized career preparation, and intensified adult identity exploration. For return migrant students, higher education becomes a powerful identity laboratory (Unzueta & Seif, 2014), an inherently transnational space characterized by constant flux and “forever becoming” (Bauman, 2012). This setting is pivotal because identity itself is a negotiated process. From a poststructuralist perspective, identity is dynamically constructed through language; each interaction provides an opportunity to renegotiate self-concept and social positioning (Weedon, 1997; Norton, 2013). Identities are therefore contingent and context-dependent rather than fixed by social structures. The identities forged in the U.S., then, are not simply transferred to Mexico. Instead, return migration catalyzes a renewed and agentive process of negotiation, with the university offering a distinctive context for adult recalibration. This experience of dislocation renders higher education an indispensable space where belonging must be consciously constructed rather than merely rediscovered.

Mora-Pablo et al. (2025) and Frausto-Hernandez (2025) show that in university, particularly in programs such as teacher education, return youth often transform negative schooling experiences into a professional, decolonial commitment to becoming more empathetic educators. Within these settings, they encounter peers with similar transnational trajectories, fostering collective advocacy for pedagogies that recognize their transnational capital. It is here that they actively perform and consolidate hybrid (Hamann & Zúñiga, 2011) or binational (Vargas, 2025) affiliations, strategically positioning themselves (Davies & Harré, 1990) through their transnational funds of knowledge (Zúñiga & Román, 2022).

Through this process, they actively construct a belonging that transcends borders. Their negotiations—whether repositioning as “100% Mexican,” adopting a supranational “Latino” identity, or reconnecting with a U.S.-influenced self (Despagne, 2024)—are agentive acts of transpositioning and therefore deliberate acts of self-definition. By focusing on return migrants who have experience as university students, this research moves beyond documenting barriers to explore how specific educational trajectories inform linguistic choices, identity negotiations, and the strategic use of multilingual repertoires.

In sum, this integrated framework posits that the three fields of scholarship reviewed are inextricably linked: the transnational structure of return creates the ideological conditions for struggles over language, within which youth exercise agency to negotiate their identities—a process that finds its most complex expression in the distinct arena of higher education. To capture the nuances of this process, the research employs a methodology that mirrors the framework’s complexity. The following section outlines a qualitative design for collecting and analyzing the narratives of return university students, aiming to trace how their multilingual repertoires and transnational biographies are strategically mobilized in the ongoing work of constructing belonging.

## 4. Methodology: A Case Study of Return Migrant Students in Mexican Higher Education

This study employed a qualitative-dominant mixed-methods approach, framed as a multi-sited case study (Yin, 2003; Stake, 1995). This design was chosen to investigate the contemporary and complex phenomenon of educational trajectories among return migrant students, allowing for an in-depth examination of the social, historical, and political influences shaping their experiences. While the number of participants (n = 26) is appropriate for an in-depth qualitative case study, the four educational profiles developed in this article should be understood as analytically constructed ideal types rather than statistically representative categories. The aim is not to generalize prevalence across the broader migrant student population, but to illuminate patterned processes of integration and identity negotiation observed within this specific institutional context.

### 4.1. Research Design and Data Collection

Data were collected in two phases: an on-site phase in 2019 at universities in Veracruz and Puebla, and a remote phase in 2021 during the COVID-19 pandemic, which enabled participation from students residing in different regions of Mexico. Both phases employed the same instruments to document the full educational trajectories central to the study’s theoretical framework. Although the shift to digital interaction may have shaped interview dynamics, preliminary coding revealed no systematic differences in core themes—such as identity negotiation, belonging, or educational aspirations—across cohorts. The two phases are therefore treated not as a comparative design but as components of a continuous qualitative inquiry.

Despite occurring under different contextual conditions, this temporal division does not introduce methodological bias. All participants had resided in Mexico prior to entering university, allowing for a more stabilized process of reintegration and a more consolidated articulation of their multiple identities, as well as a reflective awareness of their linguistic and cultural adaptation.

Twenty-six undergraduate students meeting the selection criteria (Mexican-born, with at least one year of U.S. schooling, and currently enrolled in a Mexican university) were recruited through institutional channels and snowball sampling. While this strategy may entail some visibility bias—potentially favoring students with an established institutional presence—it aligns with the study’s focus on individuals who successfully re-entered higher education following migratory trajectories. Importantly, all participants had prior socialization experiences in Mexico before university; higher education did not represent their initial contact with the national context. The study thus centers on students navigating processes of reintegration rather than those fully isolated from educational structures. The cohort was heterogeneous: ages of emigration ranged from infancy to 16, returns occurred between ages 6 and young adulthood, and several were involuntary (e.g., deportation).

Data collection integrated three instruments:

1. A demographic survey that mapped each participant’s precise educational path, enabling the coding of the core variables: age of emigration (defining generational cohort) and age of return.
2. Semi-structured interviews that explored migration histories, linguistic practices, and identity negotiations in depth.
3. Focus groups that elicited collective perspectives on adaptation and belonging.

The demographic survey also included self-assessment measures of language proficiency and national identification, which are central to the analytical framework and are described below. To examine participants’ linguistic competence in Spanish and English, the survey included a self-assessment item asking: “How would you rate your proficiency in Spanish/English?” with response options “High,” “Intermediate,” and “Low.” These categories were intentionally left open to participants’ interpretation. Rather than measuring standardized performance or formal certification, the aim was to capture their subjective perception of their linguistic repertoire across contexts of use. As such, the construct of proficiency in this study reflects perceived competence and linguistic self-positioning, not objectively tested ability.

National identification was explored through the question: “Based on your experience living in the U.S., which statement best describes you? (a) Mexican; (b) American; (c) Mexican-American; (d) None; (e) Other.” As with proficiency, these categories were appropriated and interpreted by participants themselves, allowing them to articulate their sense of belonging in their own terms. While these measures do not exhaust the processual and situated complexity of identity nor provide objective linguistic assessment, they enable comparative pattern identification across profiles in terms of perceived linguistic repertoire and reported affiliation.

### 4.2. Data Analysis

Analysis was an iterative, multi-stage process designed to connect empirical data to the theoretical framework:

1. Operationalization: Participants were categorized into generational cohorts (1.75, 1.5, 1.25) and age-of-return groups based on their surveyed educational histories.
2. Pattern Matching: Qualitative data from interviews and focus groups were analyzed to explore the theoretical propositions concerning language attrition, ideological struggle, and agentive identity negotiation. This stage specifically examined the challenges of the language-identity link and the exercise of linguistic agency.
3. Narrative Synthesis: Finally, participants’ stories were analyzed across these cohorts to understand how the specific sequence and context of their full transnational trajectory—not just emigration or return—ultimately shaped their diverse linguistic repertoires and identity configurations. This approach aligns with the framework’s emphasis on the university as a key site for adult identity negotiation.

In sum, this methodology was crafted to empirically investigate the dynamic interaction between structure (cohort, timing) and agency (linguistic practice, identity work) outlined in the theoretical framework, with a specific focus on the understudied site of higher education.

## 5. Findings

The initial analysis, segmented by generational cohort and age of return, reveals a complex landscape where linguistic and identity outcomes are shaped by a dynamic interplay of structural factors and individual agency.

### 5.1. The Conditional Influence of Generational Cohort and Age of Return

On one side, the age of emigration establishes a linguistic predisposition as suggested by previous studies (Schmid, 2008, 2013), but its effects are far from deterministic. Our data (see Table 1) reveal that generational cohorts are not monolithic; within each, significant variation concerning their language proficiency^2^ exists, pointing to the limitations of any model that relies solely on chronological benchmarks and underscoring the critical role of individual agency, which is socioculturally mediated (Ahearn, 2001).

The 1.5 Generation (emigrated at 6–12 years) emerged as the archetype of multicompetent bilingual speakers, with 6 out of 7 participants reporting high proficiency in both English and Spanish. Multicompetent speakers develop a unique integrated linguistic system with its own strength and characteristics (Cook, 1992). In participants’ cases, they had typically consolidated Spanish literacy in Mexico before undergoing immersive acquisition of English in the U.S. school system. As one 1.5-generation participant noted, *“I had the base of reading and writing in Spanish, so when I learned English in middle school, I could transfer those skills. It gave me an advantage.”* This experience aligns with the concept of sequential bilingualism—when a second language is acquired after the first one is established—and supports the analytical proposition of age of emigration within a transnational framework.

This participant’s trajectory illustrates how a specific generational cohort (Rumbaut, 2004) can successfully navigate two national education systems. The “advantage” of 1.5-generation stems from the timing of their migration, which allowed them to build academic language proficiency (Cummins, 1979) in Spanish first, a transnational fund of knowledge (Zúñiga & Román, 2022) that became a scaffold for English acquisition, ultimately facilitating multicompetence (Cook, 1992) in both languages.

Conversely, the 1.75 Generation (emigrated at 0–5 years) demonstrated a resilience of Spanish that challenges a rigid application of the language attrition theory. As Carlo shared: *“Honestly, the Spanish language wasn’t difficult for me; I picked it back up very quickly because I always practiced both English and Spanish since I was little, so it wasn’t much of a problem.”* Alan’s experience directly complicates deterministic predictions of language attrition (Schmid, 2008).

In fact, Carlo’s use of the phrase “*I always practiced it*” underscores that his Spanish was sustained not through formal instruction, but within the robust, informal ecosystem of his family. This observation qualifies a key tenet of first-language attrition theory: namely, that L1 loss following early L2 immersion is not inevitable but is contingent upon the cessation of meaningful, interactive input—input which, in Carlo’s case, was consistently provided by his family. For the 1.75 generation, the family unit becomes a crucial site of linguistic agency, actively preserving a heritage language against the odds and functioning as a bulwark against total attrition.

On the other hand, the age of return acts as a critical filter, radically reconfiguring the linguistic environment. The data reveal a clear and divergent pattern in Spanish and English proficiency based on the life stage at which individuals return (Table 2).

Spanish proficiency skews decisively toward high mastery overall (14 of 26 individuals), with the strongest outcomes for those who return very young (ages 0–5, 100% high). Crucially, low proficiency in Spanish is nearly absent (only 1 case), even among those returning as adults.

An early return (before age 13) consistently led to stronger Spanish dominance and a tendency for English attrition. Andrea’s experience is paradigmatic of this process: “*I’d say my English is at 50%... I consider that my level of English is decreasing little by little.*” This quote vividly illustrates the process of language attrition (Schmid, 2013) and reflects the power of monolingual ideologies (García & Baetens Beardsmore, 2011) within the Mexican school system. Andrea’s testimony documents an active process of L2 loss, framing it as a gradual decrease. This occurs because the Mexican educational system, permeated by a “dogma of homogeneism” (Blommaert & Verschueren, 1991), offers no support for English maintenance, effectively functioning as a powerful monolingualizing machine. Her experience shows how structural contexts can suppress transnational capital (Frausto-Hernandez, 2025), transforming a language of daily use into a vulnerable skill.

In stark contrast, English proficiency shows a strong dependence on the age of return. Those returning as adults (18+) achieved universal high proficiency (6 of 6), a pattern suggesting that English language skills solidified abroad can be preserved, specifically because it is a language with high prestige. Conversely, an early return before age 12 consistently predicted weak English outcomes, with all seven cases falling into the low or fair categories and none achieving high proficiency, despite English being a global language. The 13–17 return cohort represents a transitional group, showing a split between fair and high mastery, which may be due to how participants interpret “high” and “fair” proficiency.

While a late return safeguards English proficiency, it simultaneously challenges a common assumption: that strong English retention would correlate with weak Spanish. The data sharply contradict this. The majority of those who returned at 18+ or during adolescence (13–17) demonstrated *high* or *fair* Spanish proficiency, revealing a more complex outcome than a simple trade-off. This paradox—strong English without the expected corresponding weakness in Spanish—may be analyzed by introducing the concept of linguistic agency. This is vividly illustrated by Estela, who returned in late adolescence. Confronted with the need for academic Spanish, she declared: “*I really threw myself into studying, I took courses, I told myself I have to master this (academic Spanish)*”.

Estela’s commitment is a clear example of linguistic agency (Ahearn, 2001) and transpositioning (Wei & King Lee, 2023). Faced with an educational system offering little institutional support for her specific bilingual trajectory, she became the active architect of her own linguistic competence. Her statement represents a conscious, strategic effort to bridge a linguistic gap. This is not a story of passive loss but of active, arduous gain. By dedicating herself to study, she transpositioned her linguistic identity from a speaker of conversational Spanish to one who could master the academic registers required for success in Mexican higher education, directly resisting the institutional void she encountered.

### 5.2. Negotiated Identity: Beyond Demographic Determinism

If the analysis of language reveals conditioned patterns, the exploration of identity unveils a territory where demographic variables lose their predictive power, giving way to the forces of agency and negotiation.

The identity distribution within the 1.75 cohort disrupts the assumption that emigration in early childhood irrevocably leads to primary identification with the host country. For these young migrants, returning to Mexico acts not as a simple “return home,” but as a powerful critical event that forces a re-evaluation of pre-formed cultural affiliations. As Dani stated, *“I thought I was just American, but coming back made me have to figure out what being Mexican meant for me.”* This experience directly illustrates the concept of identity as a “forever becoming” (Bauman, 2012) and a negotiated process (Weedon, 1997; Norton, 2013). The participant’s reflection shows that their “Mexicanidad” (Mexicanity) is not an inherited trait but a reclaimed and refashioned identity. Hence, the return context functions as an identity laboratory (Unzueta & Seif, 2014), where their early U.S. socialization provides a cultural repertoire, but the act of return itself becomes the primary site for its active interpretation and re-signification.

For 1.5 and 1.25 generations, hybrid identity (“Mexican-American”) emerges as a significant force, but it is not a passive blending. Alan’s declaration, “*Regarding nationality, maybe I feel I am from both, I am Mexican-American*…” encapsulates a synthesized sense of self. His statement is a performative act of claiming a hybrid identity, a direct product of what we term *dual institutional socialization*. Having been educated in two distinct national systems, Alan internalized the cultural and linguistic resources—the transnational funds of knowledge (Zúñiga & Román, 2022)—from both. His hybridity is not a static endpoint but a continuous, introspective project.

The sheer variability of identity outcomes is illustrated by the spectrum of participant testimonies. On one end lies Irlanda’s strategic adaptability: “*If I go to the U.S., I can pass as American… and if I’m here, I’m still Mexican, so I think it’s both.*” On the other end is Evelyn’s narrative of transformative reclamation: “*I used to identify as Mexican-American… but the more I have learned, and the longer I have been here, I say yes, I am 100% Mexican*”.

Irlanda’s statement is a textbook example of transpositioning (Wei & King Lee, 2023), while Evelyn’s reflects a profound process of re-positioning (Davies & Harré, 1990) within the identity laboratory of the return context. Irlanda demonstrates a high degree of metalinguistic and metacultural agency. She treats identity not as a fixed essence but as a repertoire of linguistic and identity performances that can be strategically activated according to the demands of the environment. This is a pragmatic and agentive navigation of her transnational capital (Frausto-Hernandez, 2025). Evelyn’s journey, in contrast, shows that identity trajectories are not unidirectional. Her story involves a profound shift and re-affiliation over time, influenced by prolonged immersion and personal reflection. It demonstrates that the “self” is actively built and rebuilt through daily interactions, defying any fixed categories imposed by migration history alone.

Across cohorts, the consistent defiance of fixed identity categories reinforces a poststructuralist understanding of identity as an ongoing process of negotiation. These diverse outcomes cannot be explained by demographic variables alone, but by the interplay of educational trajectories and agentive positioning over time. The following section further examines how this dynamic unfolds in participants’ linguistic practices and institutional experiences.

### 5.3. The Impact of the Overall Educational Trajectory on Linguistic and Identity Patterns

While a separate analysis of generational cohort and age of return yields valuable insights, it also exposes their limitations. This section proposes that the complete educational sequence (as a proxy of migration trajectory for young migrants) serves as the central, unifying factor that best explains the linguistic and identity profiles of young returnees. Using ideal types, it illustrates how the interplay of primary socialization (Berger & Luckmann, 2008), formal education, and individual agency ultimately determines these outcomes.

#### 5.3.1. Profile 1: Early U.S. Immersion with Late Return

This first profile is defined by a formative educational trajectory that occurs almost entirely within the U.S. Individuals in this group completed their elementary, middle, and often high school education there, returning to Mexico for university or after finishing their secondary studies (see Table 3).

This extensive U.S. immersion results in a distinct linguistic profile. Antonio, for instance, reports “High” proficiency in all English categories but “Low” in Spanish writing and reading. In terms of identity, these individuals predominantly exhibit hybrid Mexican-American affiliations. This trajectory demonstrates how the U.S. educational system acts as a primary agent of linguistic socialization and identity formation, creating a durable foundation that a subsequent late return cannot easily overwrite. Their experience embodies the core tension of transnationalism, where affiliation and linguistic practice become decoupled from a single national territory.

Antonio’s profile is the archetype of the 1.75 generation. His linguistic data suggest that his primary socialization and literacy acquisition in English established it as his dominant language, leading to the predicted attrition of Spanish academic literacy when migrating to the U.S. However, the consistent hybrid identity across this group is the key finding. It shows that the identity laboratory of the U.S. school system was decisive, forging a lasting binational affiliation (Vargas, 2025) that a late return to Mexico does not erase. Their identity is a testament to the enduring impact of their primary institutional socialization, even within a new national context.

And yet, on the other hand, we observe cases like Carlo, who maintained exceptional Spanish proficiency not through formal schooling in Mexico, but through deliberate family policies, such as his father’s strategy of ensuring his engagement with written Spanish media: “*My dad used to read Proceso (Mexican political review) for a long time… from then on, every Sunday was about reading me all the editions.*” This consistent exposure to written Spanish via current events publications was a key familial practice that allowed him to not only preserve the language but also develop sophisticated vocabulary and sociopolitical literacy, underscoring how intentional family language planning and cultural habits can promote and ensure a high linguistic proficiency in Spanish.

Although Carlo identifies himself as a multilingual speaker (English, Spanish, French, and German), he does not feel comfortable identifying himself with a national affiliation: “*I don’t identify with a nationality; I consider that a mere bureaucratic procedure. I see myself more as a citizen of the world.*” With this statement, Carlos explicitly rejects rigid national identity categories. The concept of being a “citizen of the world” transcends the Mexican-American dichotomy, reflecting an identity shaped by multiple migratory experiences. This stance challenges nationalism as a core axis of belonging, favoring a flexible, plural affiliation shaped by global mobility over state-administered borders.

While early migration and late return structure the educational trajectory, the data suggest that these temporal patterns alone do not determine linguistic or identity outcomes. Familial language ideologies and home-based practices appear to function as decisive mediating factors, underscoring that migration timing is only one dimension within a broader ecology of socialization.

#### 5.3.2. Profile 2: Divided Secondary Socialization

Their tendency is toward the development of highly competent repertoires in both languages. Those who experienced a “both” trajectory during middle school display “High” or “Intermediate” competencies across languages, avoiding the sharp imbalances observed in the first profile (see Table 4). While we do not assume a model of balanced bilingualism—acknowledging that bilingual profiles are inherently uneven (Cook, 1992)—these findings suggest that dual-system schooling can foster advanced forms of biliteracy. Following Hornberger (2008), biliteracy encompasses fluid and multidimensional engagement with two or more languages across oral and written modalities. In identity terms, this trajectory is associated with strong hybrid affiliations: early experiences of “belonging to two worlds” become a consolidated pillar of self-understanding.

Trajectories in Table 4 embody the process of dual institutional socialization, where the sequential experience of two national school systems actively constructs a plurilithic and plurilinguistic foundation (Piccardo, 2018). These lived experiences in both educational systems make the theoretical concept of hybridity (Hamann & Zúñiga, 2011) a tangible, everyday reality.

This group, which primarily includes members of the 1.5 and 1.25 Generations, exemplifies bilingualism and hybrid identities for those who emigrated between the ages of 6 and 12. Schooling split between both systems (“both”) during basic education acts as a mechanism of secondary socialization (Berger & Luckmann, 2008) that reinforces both languages and cultures. The result, as observed in Eder, Karla, and Irlanda, is the development of multicompetence (Cook, 1992) in both languages. From the perspective of identity construction (Weedon, 1997; Norton, 2013), the experience of navigating two educational systems normalizes belonging to two worlds. This makes a hybrid identity not a contradiction, but a logical and natural outcome of their life trajectories.

For instance, Eder articulates his hybrid identity through the experience of a divided existence, stating: “*I had like two lives, no? If you look at it… I was living this type of two lives, but it was mostly because of the cultural aspects that once I had… my family and then the school*.” This quote reveals a core feature of his identity: the lived reality of a segmented, parallel life where distinct Mexican (familial, Spanish-speaking) and American (school, English-speaking) worlds operated separately. This constant negotiation between two complete cultural spheres, rather than a seamless fusion, formed the very foundation of his hybrid, yet divided, Mexican-American identity.

Eder’s high bilingual proficiency was not accidental, but the result of complementary and enforced learning contexts. His English developed through formal school instruction (ESOL) and, crucially, through active and motivated immersion in the U.S. socio-cultural and media environment, where he consciously worked to perfect it. His Spanish, however, was maintained and strengthened by a strict family language policy that designated the home as an exclusive space for Spanish, as illustrated by his mother’s rule: “*hey, aquí se habla español… stop speaking English, you need to stop and start speaking in Spanish, you’re not in school anymore*.” This clear functional division—English for public, academic, and entertainment life; Spanish for private, familial, and ancestral connection—created the ecological niches that ensured the solidity and fluidity of his competence in both languages.

However, Table 4 also reveals that linguistic multicompetence does not automatically translate into hybrid self-identification. Despite their high proficiency in English and their divergent schooling trajectories, Mireya and Karla identify exclusively as Mexican. Their cases suggest that while dual institutional socialization creates structural conditions for hybridity, identity alignment is not determined by linguistic competence alone. Factors beyond schooling—e.g., family language ideologies and affective attachments—may carry greater weight in shaping the direction of identification. Thus, hybridity emerges not as a predictable outcome of bilingualism, but as a negotiated stance within a broader ecology of socialization.

#### 5.3.3. Profile 3: Mexican Roots with Intermediate U.S. Experience

This profile is defined by a trajectory that begins with a firm educational foundation in Mexico (see Table 5). Individuals like Erwin, Ezequiel, Ashley, Priscilla, and Alan completed their primary education in Mexico, followed by a period in U.S. middle and/or high schools, before returning to Mexico for higher education or sooner.

This sequence tends to produce a distinct linguistic profile: strong proficiency in Spanish, anchored by early schooling in Mexico, combined with solid development of English during subsequent immersion. In several cases, this trajectory fosters high levels of bilingual competence, as the first language provides a cognitive and literacy foundation that facilitates transfer to the second (Cummins, 1979). However, individual outcomes vary depending on levels of personal investment and access to learning opportunities. Alan represents an intensified version of this profile: although his formal U.S. schooling was limited, his sustained and strategic investment in English^3^—through media consumption, self-directed practice, and later professional certifications—deepened his competence in both languages. His case, therefore, illustrates how structural trajectories interact with agentive investment to shape linguistic outcomes.

In terms of identity, hybrid identities are predominant in this profile, but they are built upon a solid Mexican foundation. For example, Alan clearly articulates a hybrid or bicultural identity, stating: “*I always identified a little more with American culture… because somehow I felt more welcome*.” This identity combines Mexican formal nationality with a stronger affective and cultural identification with the U.S., where he felt more accepted and socially comfortable. His identity is not a diffuse fusion, but a conscious duality where each component (the Mexican administrative/formal and the American cultural/affective) has a defined space, though his personal affinity leans toward the U.S. side.

This group, often part of the 1.25 generation, fundamentally challenges deterministic views of the critical period scholarship. Their success demonstrates that a later age of acquisition is not a barrier to high proficiency when the L1 is firmly anchored through formal schooling (Cummins, 1979; Skutnabb-Kangas, 1981). They are not passive learners but “bilinguals by agency,” who strategically leverage their U.S. immersion. Their identity negotiation is equally agentive. Unlike the innate hybridity of Profile 2, their Mexican-American identity is a conscious, post-structuralist project of the self (Weedon, 1997). It is not a primary affiliation but a deliberate expansion, where the U.S. experience is layered onto a pre-existing, solid Mexican identity, showcasing a unique form of transposition that adds a new cultural layer without displacing the core.

#### 5.3.4. Profile 4: Early Return or Brief Migration

This profile is characterized by an educational trajectory that occurs predominantly in Mexico, especially from middle school onward. The stay in the U.S. was either brief or occurred only in early childhood. This group includes individuals such as Candido, Wendy, and Andrea, as well as those with complete schooling in Mexico. Their linguistic profile shows a solid command of Spanish, while English is generally perceived as “Intermediate” or “Low,” with a few exceptions. The identity profile, however, shows greater variability, ranging from exclusively Mexican identities (e.g., Candido, Andrea, Daisy) to hybrid ones (e.g., Wendy, Evelyn) (Table 6).

This profile acts as a crucial counterfactual, demonstrating that the Mexican educational system’s monolingualizing ideology (García & Baetens Beardsmore, 2011) effectively ensures Spanish dominance in the absence of sustained countervailing forces. More importantly, it provides the clearest evidence that identity is a negotiated process (Weedon, 1997) whose outcomes cannot be predicted by structural variables alone.

The linguistic data analyzed in this paper confirm the power of institutional context: without the identity laboratory of the U.S. school system, English proficiency remains limited. However, the profound finding lies in the identity outcomes. The maximum variability here—from exclusively Mexican to hybrid—decisively refutes any linear assimilation model. For participants like Daisy, who reclaimed a “100% Mexican” identity, the return context was a site of repositioning. For others like Evelyn, maintaining a hybrid identity was an act of agentive memory and affiliation, preserving a transnational sense of self despite minimal formal U.S. schooling. Evelyn’s case, however, warrants further elaboration. Although her schooling occurred entirely in Mexico, she maintains close family ties in the U.S. and engages in short-term labor mobility there, suggesting that her hybrid identification is dynamically reconfigured through ongoing cross-border connections and anticipated mobility rather than sustained institutional immersion alone.

## 6. Discussion and Conclusions

The findings confirm that migratory timing matters—but only as a structuring condition rather than a predictive formula. Age of emigration establishes linguistic predispositions: earlier departure often correlates with stronger English and more fragile academic Spanish, whereas later emigration tends to anchor Spanish literacy. Yet these tendencies are repeatedly qualified by mediating factors. Family language policies, affective attachments, personal investment, and access to institutional support complicate generational labels. The 1.5 cohort frequently displayed multicompetence, but early emigrants (1.75) demonstrated unexpected Spanish resilience when family ecosystems sustained meaningful input. Likewise, late return often safeguarded English proficiency without producing the anticipated trade-off in Spanish, as several participants actively cultivated academic Spanish through deliberate effort. Age of return thus operates less as a deterministic threshold than as a filter that reshapes linguistic ecologies, sometimes enabling attrition, sometimes catalyzing agentive recovery. These patterns underscore a key insight: only by analyzing the complete educational trajectory—across institutions, national systems, and stages of life—can we account for the diversity of linguistic outcomes observed.

If language reveals conditioned tendencies, identity exposes the limits of demographic explanation altogether. Across cohorts, no combination of age of emigration or return reliably predicted national affiliation. Hybrid identifications emerged in structurally different profiles, while exclusively Mexican identifications appeared even among highly proficient bilinguals with divided schooling. The absence of any participant claiming an exclusively American identity, alongside cases ranging from strategic situational hybridity to explicit cosmopolitan detachment, demonstrates that belonging is not a linear extension of schooling location or linguistic dominance. Rather, identifications were articulated in a contextually situated rather than fixed manner. It is plausible that these identifications might shift under different structural conditions, such as renewed migration to the U.S. Yet, the present study focuses on return and incorporation in Mexico, underscoring that identity negotiation is relational and shaped by the institutional arenas in which it unfolds.

The findings allow us to return to the idea of the university as a new arena for identity negotiation. Across the four profiles, higher education emerges not merely as an institutional setting but as a space where prior experiences of marginalization, mobility, and adaptation are reworked. For some participants, the university represents a site of consolidation of emerging identities; for others, it becomes a terrain of tension between previous socialization and new expectations. The differentiated trajectories observed across profiles suggest that access to higher education does not homogenize experiences; instead, it reconfigures pre-existing inequalities into new forms of belonging and exclusion.

Faced with a Mexican educational system governed by a monolithic, stigmatizing ideology, return migrant students refused passive assimilation. Their response was transpositioning—the strategic navigation and transcendence of the fixed linguistic and social positions imposed by a transnational reality. Through sophisticated acts of linguistic maneuvering, they challenged established identity boundaries, created transformative new positions for themselves, and disrupted the very power structures that marginalized them. These practices were more than adaptive tactics; they were agentive acts through which these youths asserted a legitimate bilingual and bicultural belonging within a system designed to refuse it.

The university context proved essential for this transpositioning, functioning as a pivotal identity laboratory. Here, the isolated struggles of earlier schooling could be reinterpreted and transformed. For many—especially those for whom Mexico was not a return home—this space was less about rediscovering a pre-existing identity and more about actively constructing one from the ground up. Within this laboratory, personal experiences coalesced into a shared sense of transnational capital, transforming individual vulnerability into collective resourcefulness and purpose, particularly visible in fields like teacher education, where students reframe their journeys into a decolonial pedagogical commitment.

These findings call for future studies that move beyond migration timing as a primary explanatory variable and instead examine the interaction among educational trajectories, family language ideologies, affective belonging, and institutional positioning. Longitudinal research would be particularly valuable in tracing how identity identifications shift under renewed mobility or changing structural conditions. Moreover, greater attention to the university as a site of post-return identity reconfiguration could illuminate how higher education mediates the transformation of transnational capital into professional and civic engagement.

At the institutional level, universities must move beyond deficit-oriented models that interpret bilingualism and hybrid identification as transitional or problematic. Targeted policies—such as bilingual academic support, recognition of prior transnational schooling, and faculty development on raciolinguistic ideologies—are essential to prevent the reproduction of subtle exclusionary practices. More broadly, educational systems should design pedagogical frameworks that explicitly recognize transnational repertoires as legitimate academic resources rather than deviations from a monolingual norm.

Ultimately, this research presents a fundamental challenge to the Mexican educational system’s underlying monolingual and monocultural imaginary. The diverse linguistic profiles and hybrid identities documented here are not anomalous; they are the normative outcome of transnational life. The system’s failure lies in its tendency to pathologize this reality, thereby suppressing the very funds of knowledge—bilingual competence, bicultural resilience, and navigational flexibility—that constitute these students’ most significant assets. The implication is clear: moving from a paradigm of assimilation to one that actively recognizes and leverages transnational capital is not merely an inclusive ideal but an empirical necessity. It is a required step to align educational practice with the complex, agentive, and richly resourceful realities of the students it serves.

## Figures and Tables

**Table 1:** Language Proficiency by generational cohort.

Age at Emigration	Spanish			English			Total
	High	Fair	Low	High	Fair	Low	
0–5 years (Gen 1.75)	2	7	1	6	4	0	10
6–12 years (Gen 1.5)	6	1	0	6	1	0	7
13–17 years (Gen 1.25)	6	3	0	4	3	2	9
Total	14	11	1	16	8	2	26

**Table 2:** Language proficiency by age of return.

Age at Return	Spanish			English		
	Low	Fair	High	Low	Fair	High
0–5 years	0	0	4	2	2	0
6–12 years	0	1	2	0	3	0
13–17 years	0	8	5	0	6	7
18 years or more	1	2	3	0	0	6
Total	1	11	14	2	11	13

**Table 3:** Early U.S. Immersion with Late Return Trajectory.

Pseudonym	Elem-Mid-High	English Proficiency (Overall)	Spanish Proficiency (Overall)	Identity
Antonio	USA-USA-USA	High	Low	American & Mexican
Carlo	USA-USA-USA	High	High	None
Flor	USA-USA-MEX	High	Intermediate	Mexican
Vivi	USA-USA-MX	High	Intermediate	American & Mexican

The second column synthesizes the most decisive levels of education (Elementary, Middle, and High School).

**Table 4:** Profile 2: Divided secondary socialization.

Pseudonym	Elem-Mid-High	English Proficiency (Overall)	Spanish Proficiency (Overall)	Identity
Mireya	USA-BOTH-MX	High	Intermediate	Mexican
Irlanda	USA-BOTH-MX	High	Intermediate	American & Mexican
Karla	USA-BOTH-MX	High	Intermediate	Mexican
Eder	BOTH-BOTH-MEX	High	High	American & Mexican

The second column synthesizes the most decisive levels of education (Elementary, Middle, and High School).

**Table 5:** Profile 3: Mexican Roots with Intermediate U.S. Experience.

Pseudonym	Elem-Mid-High	English Proficiency (Overall)	Spanish Proficiency (Overall)	Identity
Erwin	MX-USA-USA	High	Intermediate	American & Mexican
Ezequiel	MX-BOTH-USA	High	Intermediate	Mexican
Ashley	MX-BOTH-USA	Intermediate	High	Mexican
Priscila	MX-MX-BOTH	High	Intermediate	Mexican
Alan	MX-MX-BOTH	High	High	American & Mexican

The second column synthesizes the most decisive levels of education (Elementary, Middle, and High School).

**Table 6:** Profile 4: Early return or brief migration.

Pseudonym	Elem-Mid-High	English Proficiency (Overall)	Spanish Proficiency (Overall)	Identity
Candido	USA-MX-MX	Intermediate	Intermediate	Mexican
Andrea	BOTH-MX-MX	Intermediate	High	Mexican
Daisy	MX-MX-MX	Low	High	Mexican
Wendy	BOTH-MX-MX	Intermediate	High	American & Mexican
Evelyn	MX-MX-MX	Intermediate	High	American & Mexican

The second column synthesizes the most decisive levels of education (Elementary, Middle, and High School).

## Data Availability

The data that support the findings of this study are available upon request from Mónica Jacobo and Colette Despagne. Interview transcripts are not publicly available due to ethical reasons, as they contain information that could compromise the privacy of research participants.

## Notes

1 We acknowledge that the term “proficiency” may be interpreted as aligning with hegemonic understandings of bilingualism as “two monolinguals in one” (Grosjean, 1989). However, our approach is fundamentally holistic, recognizing that bi- and plurilingual individuals possess unique linguistic profiles characterized by varying repertoires in each language they use. Accordingly, we adopt the term “proficiency” as defined in the Common European Framework of Reference for Languages (Council of Europe, 2001), which conceptualizes the language user/learner as a “social agent” who must develop specific linguistic repertoires in order to communicate effectively. The term does not imply comparison to monolingual norms nor a deficit view of bilingualism.

2 Language proficiency has been based on participants’ self-evaluation during the semi-structured interviews.

3 We draw on Norton’s (2013) concept of *investment*, understood as the learner’s socially situated commitment to language learning in pursuit of desired identities and access to symbolic and material resources.

## References

[B1-behavsci-16-00424] Ahearn L. M. (2001). Language and agency. Annual Review of Anthropology.

[B2-behavsci-16-00424] Barton D., Hamilton M. (1998). Local literacies: Reading and writing in one community.

[B3-behavsci-16-00424] Bauman Z. (2012). Liquid modernity.

[B4-behavsci-16-00424] Berger P. L., Luckmann T. (2008). La construcción social de la realidad.

[B5-behavsci-16-00424] Blommaert J., Verschueren J. (1991). The pragmatics of minority politics in Belgium. Language in Society.

[B6-behavsci-16-00424] Bustamante E. (2021). Políticas lingüísticas y educación indígena en México.

[B7-behavsci-16-00424] Cook V. J. (1992). Evidence for multicompetence. Language Learning.

[B9-behavsci-16-00424] Cortez N. A., Hamann E. T. (2014). College dreams à la Mexicana: Agency and strategy among American-Mexican transnational students. Latino Studies.

[B8-behavsci-16-00424] Council of Europe (2001). Common European framework of reference for languages: Learning, teaching, assessment.

[B10-behavsci-16-00424] Cummins J. (1979). Cognitive/academic language proficiency, linguistic interdependence, the optimum age question and some other matters. Working Papers on Bilingualism.

[B11-behavsci-16-00424] Davies B., Harré R. (1990). Positioning: The discursive production of selves. Journal for the Theory of Social Behaviour.

[B12-behavsci-16-00424] Despagne C., Zajda J., Majhanovich S. (2024). Language ideologies and identities in Mexico: The case of return migrants studying in a public Mexican university. Globalization and multicultural education.

[B13-behavsci-16-00424] Despagne C., Buitrón C., Guarda M., Stopfner M., Meier G. (2026). Plurilingual multiliteracy Spanish as a second language course for return students from the United States to Mexico. Changing perspectives towards linguistic diversity in education: Promoting social cohesion through agency.

[B14-behavsci-16-00424] Despagne C., Manzano-Mungía M. C. (2020). Youth return migration (US-Mexico): Students’ citizenship in Mexican schools. Children and Youth Services Review.

[B15-behavsci-16-00424] Escobar A., Masferrer C. (2021). La década en que cambió la migración.

[B16-behavsci-16-00424] Frausto-Hernandez I. (2025). At the intersection of transnationalism, identity and conocimiento: An autoethnography. Behavioral Sciences.

[B17-behavsci-16-00424] García O., Baetens Beardsmore H. (2011). Bilingual education in the 21st century: A global perspective.

[B19-behavsci-16-00424] González N., Moll L. C., Amanti C. (2005). Funds of knowledge. Theorizing practices in households, communities and classrooms.

[B18-behavsci-16-00424] Grosjean F. (1989). Neurolinguists, beware! The bilingual is not two monolinguals in one person. Brain and Language.

[B20-behavsci-16-00424] Hamann E. T., Zúñiga V., Vandeyar S. (2011). Schooling, national affinities, and transnational students in Mexico. Hyphenated selves: Immigrant identities within education contexts.

[B21-behavsci-16-00424] Hornberger N. H. (2008). Continua of biliteracy. Encyclopedia of language and education.

[B22-behavsci-16-00424] Jacobo M. (2017). De regreso a casa y sin apostilla: Estudiantes mexicoamericanos en México. Sinéctica Revista Educativa.

[B23-behavsci-16-00424] Jacobo M. (2022). La niñez y juventud migrante de retorno en México: Hallazgos, avances y pendientes (2015–2022). Norteamérica.

[B24-behavsci-16-00424] Jacobo M., Despagne C. (2022). Jóvenes migrantes de retorno: Construyendo nociones alternativas de ciudadanía en México. Estudios Sociológicos de El Colegio de México.

[B25-behavsci-16-00424] Jacobo M., Jensen B. (2018). When families are deported: Schooling for US-citizen students in Mexico.

[B26-behavsci-16-00424] Jardón A. E., Macías Suárez G. A. (2024). Experiencias de discriminación en el ámbito educativo de los hijos de migrantes mexicanos de retorno. Revista de Psicología de la Universidad Autónoma de México “Vulnerabilidad e Inclusión Social”.

[B27-behavsci-16-00424] Köpke B., Schmid M. S., Schmid M. S., Köpke B., Keijzer M., Weilemar L. (2004). First language attrition: The next phase. First language attrition: Interdisciplinary perspectives on methodological issues.

[B28-behavsci-16-00424] Little S., Zhou Y. (2024). Beyond roots and wings: Co-constructing a framework for heritage language children’s liminal and limbotic identities. Journal of Multilingual and Multicultural Development.

[B29-behavsci-16-00424] López R. A., Valdez Gardea G. C., Ruíz Peralta L. F., Rivera García O. B. (2018). Menores migrantes de retorno: Problemática académica y proceso administrativo en el sistema escolar sonorense. Región y Sociedad.

[B30-behavsci-16-00424] Mora-Pablo I. (2021). “Include them, don’t exclude them”: Reinsertion of return migrants in the Mexican education system. AERA Annual Meeting Conference.

[B31-behavsci-16-00424] Mora-Pablo I., Basurto Santos N. M. (2019). Experiencias educativas y de vida de migrantes de retorno: ¿Una creciente generación de maestros de inglés en México. Publicaciones.

[B32-behavsci-16-00424] Mora-Pablo I., Kasun G. S., Espinosa Z., Moyo J. N. (2025). Transnational lessons from Mexican-origin border crossing future teachers: Decolonizing teacher practices. Behavioral Sciences.

[B33-behavsci-16-00424] Nava R., Domínguez R., Castro Azuara M. C. (2017). Jóvenes universitarios repatriados: Retos y oportunidades en el área de lecto-escritura. Revista Paradigma.

[B34-behavsci-16-00424] Norton B. (2013). Identity and language learning: Extending the conversation.

[B35-behavsci-16-00424] Paris M. D., Hualde A., Woo O. (2019). Experiencias de migrantes mexicanos en contextos urbanos.

[B36-behavsci-16-00424] Parra M. L., Rodríguez Cruz M., Rodríguez-Cruz M., Vázquez J. D. (2025). Mediación intercultural y los procesos de (re)inserción escolar en población migrante transnacional y de retorno: Una propuesta para el caso de Oaxaca. Niñez, adolescencia y juventud migrante en las Américas: Análisis y narrativas de una movilidad emergente.

[B37-behavsci-16-00424] Piccardo E., Trifonas P. P., Aravossitas T. (2018). Plurilingualism: Vision, conceptualization, and practices. Handbook of research and practice in heritage language education.

[B38-behavsci-16-00424] Rodríguez-Cruz M. (2022). Stereotype and stigma in the school (re)insertion of ‘children of deported parents’ from the United States: An analysis in Oaxaca, Mexico. Latino Studies.

[B39-behavsci-16-00424] Rosa J., Flores N. (2017). Unsettling race and language: Toward a raciolinguistic perspective. Language in Society.

[B40-behavsci-16-00424] Ruíz L. F., Valdez G. C. (2024). Estrategias directivas para el ingreso de niñas, niños y adolescentes migrantes retornados en las escuelas de Sonora. La aplicabilidad de las normas de control escolar. Región y Sociedad.

[B41-behavsci-16-00424] Rumbaut R. G. (1994). The crucible within: Ethnic identity, self-esteem, and segmented assimilation among children of immigrants. International Migration Review.

[B42-behavsci-16-00424] Rumbaut R. G. (2004). Ages, life stages, and generational cohorts: Decomposing the immigrant first and second generations in the United States. International Migration Review.

[B43-behavsci-16-00424] Schmid M. S. (2008). Defining language attrition. Babylonia.

[B44-behavsci-16-00424] Schmid M. S. (2011). Language attrition and identity.

[B45-behavsci-16-00424] Schmid M. S. (2013). First language attrition. Linguistic Approaches to Bilingualism.

[B46-behavsci-16-00424] Skerrett A. (2020). Teaching transnational youth: Literacy and education in a changing world.

[B47-behavsci-16-00424] Skutnabb-Kangas T. (1981). Bilingualism or not. The education of minorities.

[B48-behavsci-16-00424] Smith M. P. (1998). Transnationalism from below: Comparative urban and community research.

[B49-behavsci-16-00424] Stake R. E. (1995). The art of case study research.

[B50-behavsci-16-00424] Tang F., Calafato R. (2025). Family language policy: The impact of multilingual experiences at university and language practices at home. International Journal of Bilingual Education and Bilingualism.

[B51-behavsci-16-00424] Trejo N. P., Mora Vázquez A., Andrade Rubio K. L. (2023). Navigating the transition from the American into the Mexican educational system. Bilingual Research Journal.

[B52-behavsci-16-00424] Trejo N. P., Mora Vázquez A., Mora-Pablo I., Lengeling M., Crawford T. (2016). Learning transitions of returnee English language teachers in Mexico. Lenguas en Contexto.

[B53-behavsci-16-00424] Umaña-Taylor A. J., Quintana S. M., Lee R. M., Cross W. E., Rivas-Drake D., Schwartz S. J., Syed M., Yip T., Seaton E., Ethnic and Racial Identity in the 21st Century Study Group (2014). Ethnic and racial identity during adolescence and into young adulthood: An integrated conceptualization. Child Development.

[B54-behavsci-16-00424] Unzueta T., Seif H. (2014). Disrupting the dream: Undocumented youth reframe citizenship and deportability through anti-deportation activism. Latino Studies.

[B55-behavsci-16-00424] Vargas E., Giorguli S., Bautista A. (2022). Los desafíos para la integración escolar de los migrantes de Estados Unidos a México. Derechos fragmentados. Acceso a derechos sociales y migración de retorno a México.

[B56-behavsci-16-00424] Vargas E. (2025). National identifications of transnational students from the USA of the northwest of Mexico. International Migration.

[B57-behavsci-16-00424] Vasconcelos J. (1948). La raza cósmica: Misión de la raza iberoamericana.

[B58-behavsci-16-00424] Weedon C. (1997). Feminist practice and poststructuralist theory.

[B59-behavsci-16-00424] Wei L., King Lee T. (2023). Transpositioning: Translanguaging and the liquidity of identity. Applied Linguistics.

[B60-behavsci-16-00424] Yin R. K. (2003). Case study research: Design and methods.

[B61-behavsci-16-00424] Zúñiga V. (2025). International migration and linguistic vicissitudes. Linguistic Approaches to Bilingualism.

[B62-behavsci-16-00424] Zúñiga V., Hamann E. T. (2008). Escuelas nacionales, alumnos transnacionales: La migración México/Estados Unidos como fenómeno escolar. Estudios Sociológicos.

[B63-behavsci-16-00424] Zúñiga V., Román B. (2022). Maestros de las escuelas de México: Competencias docentes necesarias para atender a las alumnas y los alumnos procedentes de Estados Unidos. Abordajes interdisciplinarios sobre la niñez y la adolescencia migrante.

